# Whole-Genome Sequences of Two Carbapenem-Resistant *Klebsiella quasipneumoniae* Strains Isolated from a Tertiary Hospital in Johor, Malaysia

**DOI:** 10.1128/genomeA.00768-17

**Published:** 2017-08-10

**Authors:** Han Ming Gan, Ganeswrie Rajasekaram, Wilhelm Wei Han Eng, Priyatharisni Kaniappan, Amreeta Dhanoa

**Affiliations:** a Genomics Facility, Tropical Medicine and Biology Platform, Monash University Malaysia, Bandar Sunway, Petaling Jaya, Selangor, Malaysia; b School of Science, Monash University Malaysia, Bandar Sunway, Petaling Jaya, Selangor, Malaysia; c School of Life and Environmental Sciences, Deakin University, Geelong, Victoria, Australia; d Department of Pathology, Hospital Sultanah Aminah Johor Bahru, Johor Bahru, Johor, Malaysia; e Jeffrey Cheah School of Medicine and Health Sciences, Monash University Malaysia, Bandar Sunway, Selangor, Malaysia

## Abstract

We report the whole-genome sequences of two carbapenem-resistant clinical isolates of *Klebsiella quasipneumoniae* subsp. *similipneumoniae* obtained from two different patients. Both strains contained three different extended-spectrum β-lactamase genes and showed strikingly high pairwise average nucleotide identity of 99.99% despite being isolated 3 years apart from the same hospital.

## GENOME ANNOUNCEMENT

Carbapenem-resistant *Enterobacteriaceae*, which include *Klebsiella pneumoniae*, are resistant to almost all currently available antibiotics and pose an urgent threat to global human health (1). Three main phylogenetic lineages of *Klebsiella pneumoniae* had been previously identified, KpI, KpII, and KpIII (2). However, subsequent taxonomic studies have further subdivided phylogroup KpII to *K. quasipneumoniae* subsp. *quasipneumoniae* and *K. quasipneumoniae* subsp. *similipneumoniae*, corresponding to two subgroups, KpII-A and KpII-B, respectively (2). Invasive human infections with *K. quasipneumoniae* have been reported (3).

Strains MGF005 and MGF008 were isolated from clinical specimens of two different patients (3 years apart) in a tertiary hospital located at Johor Bharu, Johor, Malaysia. Genomic DNA extraction was performed from a 2-day-old nutrient agar culture using the Sokolov method (4). The purified genomic DNA (gDNA) was quantified using Qubit BR (Invitrogen, Carlsbad, CA) and tagmented with Nextera XT (Illumina, San Diego, CA). The constructed libraries were then sequenced in two MiSeq runs (2- × 150- and 2- × 250-bp run settings). Nextera adapter trimming was performed using SeqPurge and the trimmed reads were subsequently error corrected with BFC (5, 6). The adapter-trimmed and error-corrected paired-end reads were assembled using SPAdes version 3.10 in the careful mode (7). The average nucleotide identity was calculated using the online version of JSpecies (8), and the identification of antibiotic resistance genes was performed with SSTAR version 1.1.01 (90% cutoff sequence similarity value for new variants) utilizing the ARG-ANNOT database (accessed 12 July 2016) (9, 10).

SPAdes assembly resulted in final assemblies of 5,378,118 bp (*N*_50_ of 474,846 bp, GC content of 57.78%, and 47 contigs) and 5,376,081 bp (*N*_50_ of 476,434 bp, GC content of 57.78%, and 50 contigs) for strains MGF005 and MGF008, respectively. Both strains exhibit a pairwise average nucleotide identity of 99.23% to *Klebsiella quasipneumoniae* subsp. *similipneumoniae* DSM 28212^T^(2). Interestingly, both strains exhibit a strikingly high average nucleotide identity of 99.99% between each other despite having been isolated 3 years apart. Consistent with their high genomic similarity, the strains share similar antibiotic resistance gene profiles. Apart from colistin, both MGF005 and MGF008 were found to be resistant to all antibiotics tested according to CLSI procedures for disk diffusion (11). These antibiotics included cephalosporins (cephalexin, cefuroxime, ceftazidime, ceftriaxone, cefotaxime, and cefepime), β-lactam/β-lactamase inhibitors (ampicillin-sulbactam, piperacillin-tazobactam, and amoxicillin-clavulanic), carbapenems (imipenem, meropenem, and ertapenem), aminoglycosides (gentamicin and amikacin), ciprofloxacin, tigecycline, and trimethoprim-sulfamethoxazole. Notably, the presence of three different *bla* genes (*bla*_CTX-M-14_, *bla*_TEM-1D_, and *bla*_NDM-5_) that code for extended-spectrum β-lactamase (12) in both genomes corroborates their observed antibiotic resistance to all cephalosporins, β-lactam/β-lactamase inhibitors, and carbapenems, respectively. Both strains were susceptible to colistin, with an MIC of 0.5 μg/ml. Carbapenem MIC determination using the Etest (11) was performed for MGF005 and showed MICs of 32 μg/ml (imipenem and ertapenem) and 8 μg/ml (meropenem). To the best of our knowledge, this is the first report of whole-genome sequences of *Klebsiella quasipneumoniae* subsp. *similipneumoniae* from clinical isolates that harbor carbapenem resistance genes.

### Accession number(s).

The whole-genome shotgun projects for strains MGF005 and MGF008 have been deposited in DDBJ/EMBL/GenBank under the accession numbers NEWB00000000 and NEWC00000000, respectively. The versions described in this paper are the first versions.
